# The risk of venous thromboembolism in kidney transplant recipients is enhanced following a cytomegalovirus infection

**DOI:** 10.3389/fimmu.2026.1816608

**Published:** 2026-05-08

**Authors:** Christophe Masset, Marine Lorent, Florent Le Borgne, Anne Scemla, Clarisse Kerleau, Celine Bressollette-Bodin, Emmanuel Morelon, Xavier Charmetant, Moglie Lequintrec-Donnette, Vincent Pernin, Marc Ladrière, Sophie Girerd, Olivier Aubert, Christophe Legendre, Antoine Sicard, Laetitia Albano, Carmen Lefaucheur, Gillian Divard, Christophe Mariat, Guillaume Claisse, Jacques Dantal, Magali Giral

**Affiliations:** 1 Institut de Transplantation Urologie Néphrologie (ITUN), Centre Hospitalier Universitaire (CHU) Nantes, Nantes, France; 2 Center for Research in Transplantation and Translational Immunology, Nantes Université, INSERM, UMR 1064, Nantes, France; 3 INSERM UMR 1246 - SPHERE, Nantes Université, Tours Université, Nantes, France; 4 Service de Néphrologie - Transplantation rénale Adulte, Hôpital Necker-Enfants Malades, Assistance Publique-Hôpitaux de Paris, Université Paris Descartes Sorbonne Paris Cité, Paris, France; 5 Hospices Civils de Lyon, Hôpital Edouard Herriot, Service de Transplantation, Néphrologie et Immunologie Clinique, Lyon, France; 6 Department of Nephrology and Renal Transplantation, Lapeyronnie Hospital and INSERM U1183, Institute of Regenerative Medicine and Biotherapies, Montpellier, France; 7 Renal Transplantation Department, Brabois University Hospital, Nancy, France; 8 Nephrology, Dialysis and Transplantation Department, Pasteur 2 Hospital, Nice University Hospital, Nice, France; 9 Department of Nephrology and Kidney Transplantation, Saint-Louis Hospital, Assistance Publique-Hôpitaux de Paris, Paris, France; 10 Service de Néphrologie, Dialyse et Transplantation Rénale, Hôpital Nord, Centre Hospitalier Universitaire (CHU) de Saint-Etienne, Saint Etienne, France

**Keywords:** CMV, immunothrombosis, kidney transplantation, pancreas - kidney transplantation, thrombosis

## Abstract

**Introduction:**

In immunocompetent individuals, past CMV infection has been linked to venous thromboembolism (VTE) risk, but evidence remains inconclusive due to timing of serologic diagnosis. Data in immunocompromised patients are scarce. We therefore assessed VTE risk following CMV viremia in kidney transplant recipients (KTRs).

**Methods:**

We conducted a multicenter cohort study of 15, 433 KTRs transplanted between 2000 and 2021, among whom 1, 756 developed CMV infection, with a 2-year cumulative incidence of 13% (95% CI: 12.4–13.5%). VTE occurred in 6.7% of KTRs (95% CI: 6.3–7.1%).

**Results:**

Using time-varying Cox models stratified on centers and transplant period, asymptomatic CMV viremia (considered as a time-dependent parameter) was significantly associated with an increased VTE risk (HR = 1.61, 95% CI: 1.19–2.17), with a higher risk after symptomatic CMV disease (HR = 2.00, 95% CI: 1.32–3.02), after adjustment for confounders. The risk was similar following primary infection or a reactivation of latent CMV.

**Conclusion:**

In conclusion, CMV viremia, especially symptomatic disease, was associated to a higher risk of VTE in KTRs. Given the frequency of CMV post-transplant, clinicians should be aware of this association for prompt diagnosis and management.

## Introduction

Venous thromboembolism (VTE), including deep vein thrombosis (DVT) and pulmonary embolism (PE) affects more than 10 million people per year worlwide (1). Multiple well-known risk factors have been described including age (2), hormonal factors (3), specific genetic mutations (4), malignancies (5), obesity (6) and the post-surgical period. Infectious diseases may also be related to the occurrence of VTE due to the inflammatory mechanism described as *immunothrombosis (*7, 8). Recently, the Covid-19 pandemic has highlighted the specific risk of thrombosis due to systemic inflammation related to infectious diseases (9).

Cytomegalovirus (CMV) is a DNA virus from the Herpesviridae family. Whilst infection is largely widespread, it is generally pauci-symptomatic in the large majority of immunocompetent people. In immunocompromised people, and especially organ transplant recipients, CMV infection can lead to serious complications such as organ involvement (CMV disease) (10). Besides, it also lead to immune modulation, impacting both immunosuppression burden (either directly or linked to the decrease in drugs by the transplant physician (11)) and activation of the immune system, particularly alloimmune reaction (12, 13). Several years ago, VTE was described as a rare complication of CMV infection in immunocompetent patients (14–16). However, despite multiple reports, the correlation between CMV infection and VTE has been difficult to certify, mainly because of the uncertainty in establishing the exact timing of CMV infection in immunocompetent patients. Indeed, in the general population CMV diagnosis is performed using serological assessment, which carries several biases including the long term persistence of anti-CMV IgM (17, 18), but also possible false positive IgM serology (19, 20).

In kidney transplantation, CMV viremia is routinely and regularly monitored due to the well-known increased risk of CMV viremia during the early post-transplantation period (10). However, to date, reports of VTE consecutive to CMV infection are limited to only a few case reports (21–23).

The objective of our study was to determine the specific risk of VTE occurrence following CMV viremia using a large cohort of kidney transplant recipients.

## Methods

### Studied population

We conducted a multi-center study on all consecutive adult patients transplanted with a kidney alone, or a combined kidney–pancreas in the French university hospitals of Lyon, Montpellier, Nantes, Nancy, Nice, Paris (Saint Louis and Necker) and Saint-Etienne between January 1^st^, 2000, and August 31^st^, 2021. Data collection for Lyon, Nice and Saint-Etienne started in 2006, 2013 and 2018 respectively. All data were extracted from the observational and prospective database of the cohort of transplanted patients of the French DIVAT Network (www.divat.fr).

### Available data

Recipient characteristics were age, gender, body mass index (BMI), transplantation rank, initial renal disease (possibly recurrent or not), history of cardiovascular disease and hypertension, history of VTE, history of malignancy, and pretransplant anti-human leukocyte antigen (HLA) immunization. Donor features were age, gender, and donor type: living or deceased; from a standard criteria donor (SCD) or an expanded criteria donor (ECD). Recipient and donor CMV serology were retained, as well as the use of CMV prophylaxis (valganciclovir). Baseline transplantation parameters were transplantation type (kidney or kidney and pancreas), number of HLA A-B-DR incompatibilities and induction therapy (T cell depleting or not). Post-transplantation parameters were occurrence of delayed graft function (at least one dialysis within the first seven days), the first occurrence of a CMV viremia, surgical complications (post-operative complication requiring re-operation), and VTE (including pulmonary embolism and deep vein thrombosis), and finally the use of maintenance immunosuppressive therapy. Protocols regarding CMV management and prevention of VTE complications after transplantation depending on centers is provided in **Supplementary Tables 1**–**5**. The patient follow-up and data collection stopped upon return to dialysis, re-transplantation, death, or loss to follow-up for at least 2 years.

### Characterization of CMV and VTE events

CMV infection was considered as either the occurrence of an asymptomatic viremia (≥2.5 log; “*CMV DNAemia*”); or a CMV Syndrom or CMV Disease grouped as “*Symptomatic CMV*” during the post-transplantation follow-up (24). Of note, most patients in our cohort received a prophylactic strategy regarding donor and recipient CMV serological status. Both primary infection and reactivation were considered in the main analysis. A further interaction analysis was conducted to investigate a possible difference between those situations. In patients presenting a CMV infection, we retrospectively collected biological information regarding the total duration of CMV viremia when available.

VTE events included all acute pulmonary embolism and deep vein thrombosis events during the post-transplantation follow-up diagnosed by ultrasound or CT-scan. Chronic thrombotic complications, superficial venous thrombosis and allograft thrombosis were not considered as VTE in our analysis. A standardized prevention of VTE episodes was performed in the immediate post-transplant time in most centers. In the main analysis, only VTE occurring after a CMV infection were considered. We included both incidental and for cause VTE diagnosis.

### Statistical analyses.

The characteristics at transplantation were described using frequency and proportion for categorical variables and mean and standard deviation for continuous variables. Even though CMV is time-dependent we proposed a description according to two groups: patients who had not had CMV infection and patients with at least one CMV infection in the two years post-transplantation. Because these exposure groups are time-dependent, we did not provide statistical tests. The outcome was the VTE-free survival time, defined by the time between transplantation and the first occurrence of VTE. Participants alive with a functioning graft and without VTE were censored at 2-years post-transplantation. The cumulative incidence curves of the VTE and CMV were obtained by the Aalen-Johansen estimator, considering death and/or graft failure as competing events (25). Multivariable cause-specific time-varying Cox models stratified on centers and period of transplantation were used to estimate the relationship between the incidence of CMV and the hazard of VTE, with CMV included as a time-dependent variable (26). Competing events were right-censored (27). The time dependent variable for CMV infection included in our analysis allows each patient to be in the CMV-free group at the time of transplantation and to then move and remain into the CMV group at the time we observed a CMV for the first time for the patient. In a complementary analysis we distinguished the asymptomatic DNAemia to the symptomatic CMV. The hazard proportionality assumption was tested from the Schoenfeld residuals (28). If this assumption did not hold, two different periods were considered. For baseline continuous covariates, the log-linearity assumption was checked by univariate analysis if the Bayesian Information Criterion was not reduced using natural spline transformation compared to the inclusion of the covariate in its natural scale. In case of violation, variables were categorized. We included surgical complications post-transplantation as a time-dependent covariate in our cause-specific time-varying Cox models but did not consider surgical complications occurring after CMV as it is a mediator in the association between CMV and VTE (29). Significant variables in the unadjusted analysis (p < 0.20) were included in the multivariate model. We tested interactions between CMV and three baseline covariates: history of cardiovascular disease, history of VTE and recipient CMV serology. Interactions were retained in the multivariable model if statistically significant. Patients with missing data on the covariates retained in the multivariable models were excluded. We described the characteristics of the studied patients and those of the excluded patients. To visually represent the time-dependent association between CMV viremia and VTE occurrence, we used a Simon-Makuch plot, which accounts for the time-dependent nature of CMV exposure. Unlike standard Kaplan-Meier curves, this approach dynamically reclassifies patients from the CMV-free group to the CMV group at the exact time of their first CMV viremia, thereby avoiding immortal-time bias. Cumulative incidence of VTE was estimated and plotted separately for each exposure group over the 2-year post-transplantation follow-up period. Because some post-transplant complications may influence immunosuppressive drug prescription, we analyzed their impact on the CMV – VTE association. We focused specifically on mTOR inhibitors and steroids because these may be related to risk factors of VTE. Due to the low event number and multiple changes in immunosuppressive drugs during the follow-up, this data remained only descriptive for this analysis.

We used R version 3.6.1 (30) for all data analyses.

### Ethical consent

All consecutive patients were included, and data was extracted from the DIVAT database. This study received data privacy approval from CNIL (09-17-2004, number n°891735, Réseau DIVAT:10.16.618). The patient’s non-opposition regarding access to their medical records, collection and data processing is mandatory under French law. All data were anonymized before analysis. The quality of the DIVAT data bank is validated by an annual audit. The clinical and research activities being reported are consistent with the Principles of the Declaration of Istanbul as outlined in the Declaration of Istanbul on Organ Trafficking and Transplant Tourism.

## Results

### Recipient demographic characteristics

**Figure 1:**
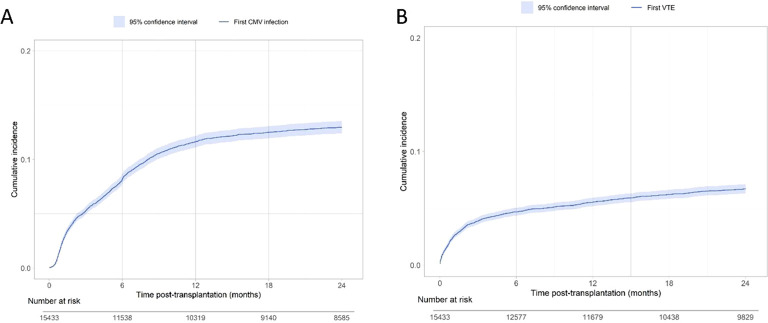
**(A)** Cumulative incidence curve of first occurrence of CMV infection. **(B)** Cumulative incidence curves of venous thromboembolism after transplantation (Aalen-Johansen estimator, returns to dialysis and deaths as competing events).

We included 15, 433 recipients of a kidney (n= 14, 510) or combined kidney/pancreas transplant (n= 923). The characteristics of the patients according to CMV infection are described in **Table 1**. Among participants, 1, 756 had at least one CMV infection in the two-years following transplantation and 967 had a history of VTE before transplantation. 58.2% of recipients with a positive CMV serology received a 3-months antiviral prophylaxis, and 88.6% of the recipients with a negative CMV serology and positive donor serology received a 6-months antiviral prophylaxis, **Supplementary Tables 1**–**4**. The cumulative incidence curve of the first CMV infection is presented in **Figure 1a**. The probabilities of having at least one CMV infection at 1- and 2-years post-transplantation were 11.6% (95% confidence interval (CI) from 11.1% to 12.2%) and 13.0% (95% CI from 12.4% to 13.5%), respectively. During the same period 956 patients presented a VTE, 907 patients returned to dialysis or were re-transplanted and 479 died with a functioning graft.

**Table 1:** Characteristics of the 15, 433 transplant recipients included in the analysis according to the occurrence of CMV infection during the follow-up of two-years post-transplantation.

Parameter	Whole sample (n=15, 433)			No CMV infection (n=13, 677)			At least one CMV infection in the 2 years post-transplantation (n=1, 756)		
	NA	n	%	NA	n	%	NA	n	%
**Male sex**	0	9610	62.3	0	8545	62.5	0	1065	60.6
**Re-transplantation**	0	2596	16.8	0	2347	17.2	0	249	14.2
**Kidney transplantation**	0	14510	94.0	0	12862	94.0	0	1648	93.8
**Recurrent initial nephropathy**	0	4144	26.9	0	3695	27.0	0	449	25.6
**History of cardiovascular disease**	0	5935	38.5	0	5157	37.7	0	778	44.3
**History of hypertension**	0	12583	81.5	0	11143	81.5	0	1440	82.0
**History of venous thromboembolism**	0	967	6.3	0	850	6.2	0	117	6.7
**History of malignancy**	0	1636	10.6	0	1436	10.5	0	200	11.4
**Positive recipient CMV serology**	0	10028	65.0	0	8920	65.2	0	1108	63.1
**Male donor**	16	8790	57.0	16	7846	57.4	0	944	53.8
**Donor type**	160			141			19		
*ECD*		4956	32.4		4208	31.1		748	43.1
*Living*		2367	15.5		2163	16.0		204	11.7
*SCD*		7950	52.1		7165	52.9		785	45.2
**Positive donor CMV serology**	0	8239	53.4	0	6894	50.4	0	1345	76.6
**HLA-A-B-DR mismatches > 4**	242	6897	45.4	214	6056	45.0	28	841	48.7
**Depleting induction**	97	9074	59.2	95	7798	57.4	2	1276	72.7
**CMV prophylaxis at transplantation**	607	9535	64.3	567	8419	64.2	40	1116	65.0
**Positive anti HLA class I**	2190	5179	39.1	1927	4549	38.7	263	630	42.2
**Positive anti HLA class II**	2902	4930	39.3	2612	4347	39.3	290	583	39.8
**Delayed graft function**	846	3567	24.5	810	3114	24.2	36	453	26.3
**Surgical complication**	0	4256	27.6	0	3635	27.2	0	621	30.0
**At least one CMV infection**	0	1756	11.4	0	0	0.0	0	1756	100.0
	**NA**	**m**	**SD**	**NA**	**m**	**SD**	**NA**	**m**	**SD**
**Recipient age (years)**	0	50.1	14.2	0	49.7	14.2	0	53.2	14.1
**Recipient BMI (kg/m²)**	136	24.5	4.4	124	24.4	4.4	12	25.0	4.5
**Donor age (years)**	49	50.9	16.4	41	50.4	16.4	8	55.0	16.1

BMI, body mass index; CMV, cytomegalovirus; ECD, expanded criteria donors; HLA, human leucocyte antigens; NA: not available (missing); SCD, standard criteria donors; SD, standard deviation.

### Risk factors of venous thromboembolism in kidney transplant recipients

**Figure 2:**
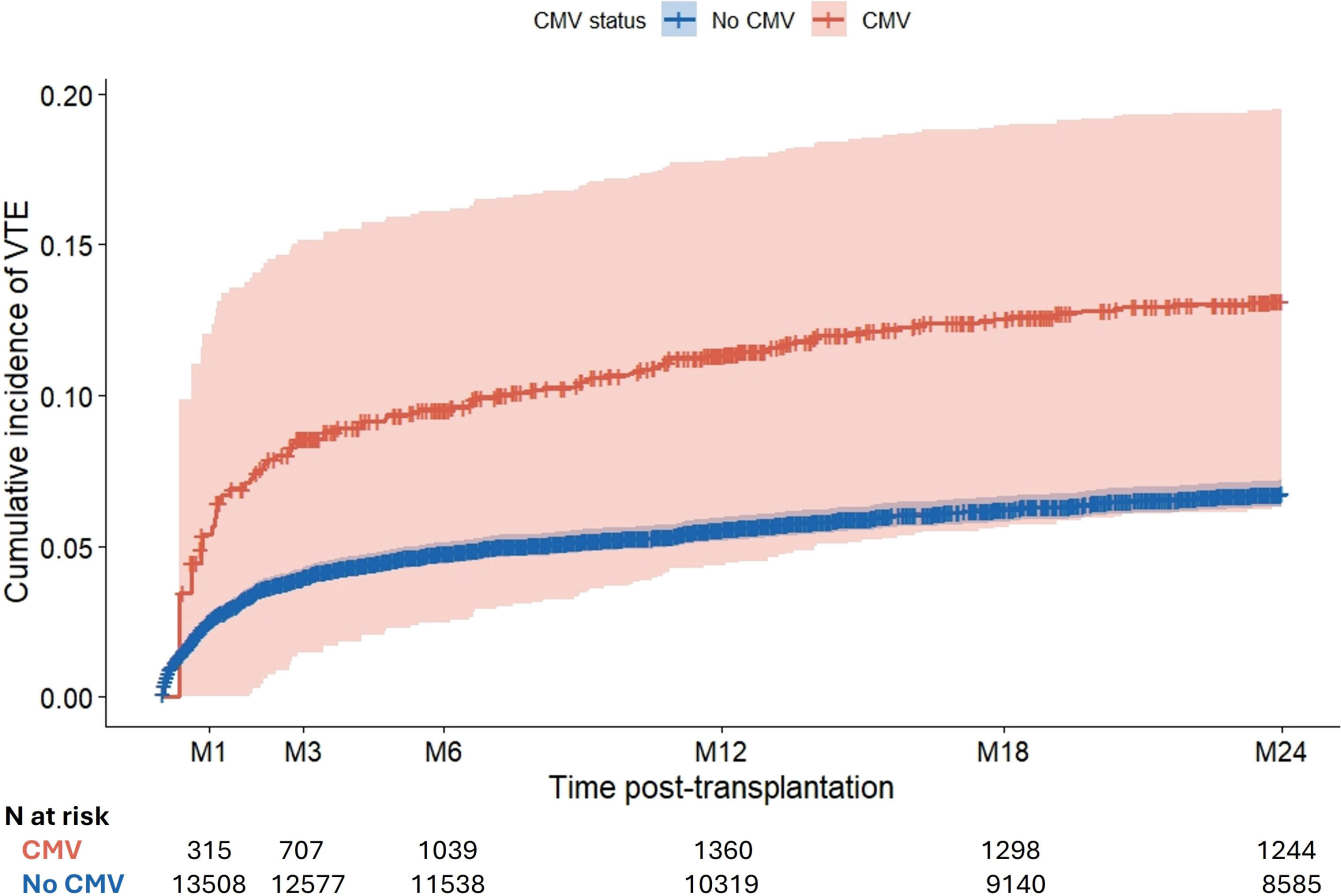
Simon-Makuch plot of VTE cumulative incidence according to CMV status in kidney transplant recipients. Cumulative incidence of venous thromboembolism (VTE) is shown for patients with (red) and without (blue) CMV viremia over the 2-year post-transplantation follow-up. CMV status is treated as a time-dependent exposure: patients are initially classified in the CMV-free group and dynamically reassigned to the CMV group at the time of their first documented CMV viremia. Shaded areas represent 95% confidence intervals. CMV: cytomegalovirus; VTE: venous thromboembolism.

The cumulative incidence rates of a first VTE at 1- and 2-years post-transplantation was 5.53% (95% CI from 5.17% to 5.92%) and 6.71% (95% CI from 6.31% to 7.14%), respectively (**Figure 1b**). Results of the unadjusted Cox model is presented in **Supplementary Table 6**. For the multivariable analysis, 1, 490 patients were excluded due to missing data concerning covariates. Their characteristics are described in **Supplementary Table 7** and showed no demographic difference to the retained 13, 943 patients included in the final multivariable cause specific time varying Cox model presented in **Table 2**. Four factors were independently associated with an increased risk of post-transplant VTE: recipient age (HR = 1.01; CI 95% [1.00; 1.02]), history of VTE before transplantation (HR = 2.46; CI 95% [2.00; 3.03]), occurrence of a surgical complication (HR 1.60; CI 95% [1.38; 1.87]), and CMV DNAemia (HR = 1.61; CI 95% [1.19; 2.17]), **Table 2**; **Figure 2**. Interestingly, the risk associated with CMV infection was even higher in the case of symptomatic CMV (HR = 2.00; CI95% [1.32; 3.02]). We also noticed a trend to a higher risk due to history of recipient malignancy (HR = 1.19; CI95% [0.98;1.45]) and retransplantation (HR = 1.18; CI95% [0.99; 1.41]). Of note, the interaction between the recipient CMV serology at transplantation and the CMV infection was not retained in the final multivariable model indicating that we did not observe an increased risk of VTE after a CMV infection for patients with a positive CMV serology at transplantation compared to patients with negative CMV serology. This result was consistent with sensitivity analyses demonstrating similar associations in both R+ and D+/R- subgroups (**Supplementary Tables 8**, **9**), as well as in the kidney alone transplant subgroup (**Supplementary Table 10**). Similarly, the results remained unchanged when considering surgical complications that may have occurred after the CMV infection (**Supplementary Table 11**). In other words, CMV primary infection and CMV reactivation lead to a similar risk of VTE post-transplantation.

**Table 2:** Results of the multivariable cause-specific time-dependent Cox model stratified on centers studying the risk of venous thromboembolism in the first two years post-transplantation (n=13, 943, 1, 490 recipients were removed because of missing data, n=956 VTE occurred after transplantation among which 87 followed a first CMV infection).

Parameter	HR	95% CI	p-value
**CMV infection**			0.0002
*CMV DNAemia*	1.62	[1.20; 2.19]	
*Symptomatic CMV*	1.97	[1.31; 2.98]	
**Year of transplantation > 2010**	0.84	[0.73; 0.96]	0.0132
**Recipient age (years)**	1.01	[1.00; 1.02]	0.0009
**Recipient BMI (kg/m²)**	1.01	[1.00; 1.03]	0.1088
**Retransplantation**	1.18	[0.99; 1.41]	0.0624
**History of cardiovascular disease**	1.03	[0.88; 1.20]	0.7193
**History of hypertension**	1.10	[0.91; 1.33]	0.3205
**History of venous thromboembolism**	2.50	[2.03; 3.08]	<0.0001
**History of malignancy**	1.21	[1.00; 1.47]	0.0558
**Donor age (years)**	1.00	[0.99; 1.01]	0.7114
**Donor type**			0.4144
**ECD (vs. Living)**	0.93	[0.73; 1.17]	
**SCD (vs. Living)**	0.90	[0.73; 1.11]	
**HLA-A-B-DR mismatches > 4**	1.12	[0.98; 1.28]	0.1092
**CMV prophylaxis treatment at transplantation**	1.15	[0.97; 1.37]	0.1043
**Delayed graft function**	1.09	[0.93; 1.27]	0.2818
**Surgical complication**	1.57	[1.35; 1.84]	<0.0001

BMI, body mass index; CI, confidence interval; CMV, cytomegalovirus; ECD, expanded criteria donors; HLA, human leucocyte antigens; HR, hazard ratio; SCD, standard criteria donors; SD, standard deviation.

### Timing of venous thromboembolism diagnosis in relation to onset of CMV viremia

Among the 1, 756 KTR who presented a CMV infection during the first two years post-transplantation, VTE occurred in 10.2% of them meaning that 179 KTR had both CMV and VTE complications during the first two-years post transplantation. Among these, 87 presented a VTE after CMV diagnosis (which corresponds to the ones retained in our multivariable cause-specific time varying Cox model).

**Figure 3:**
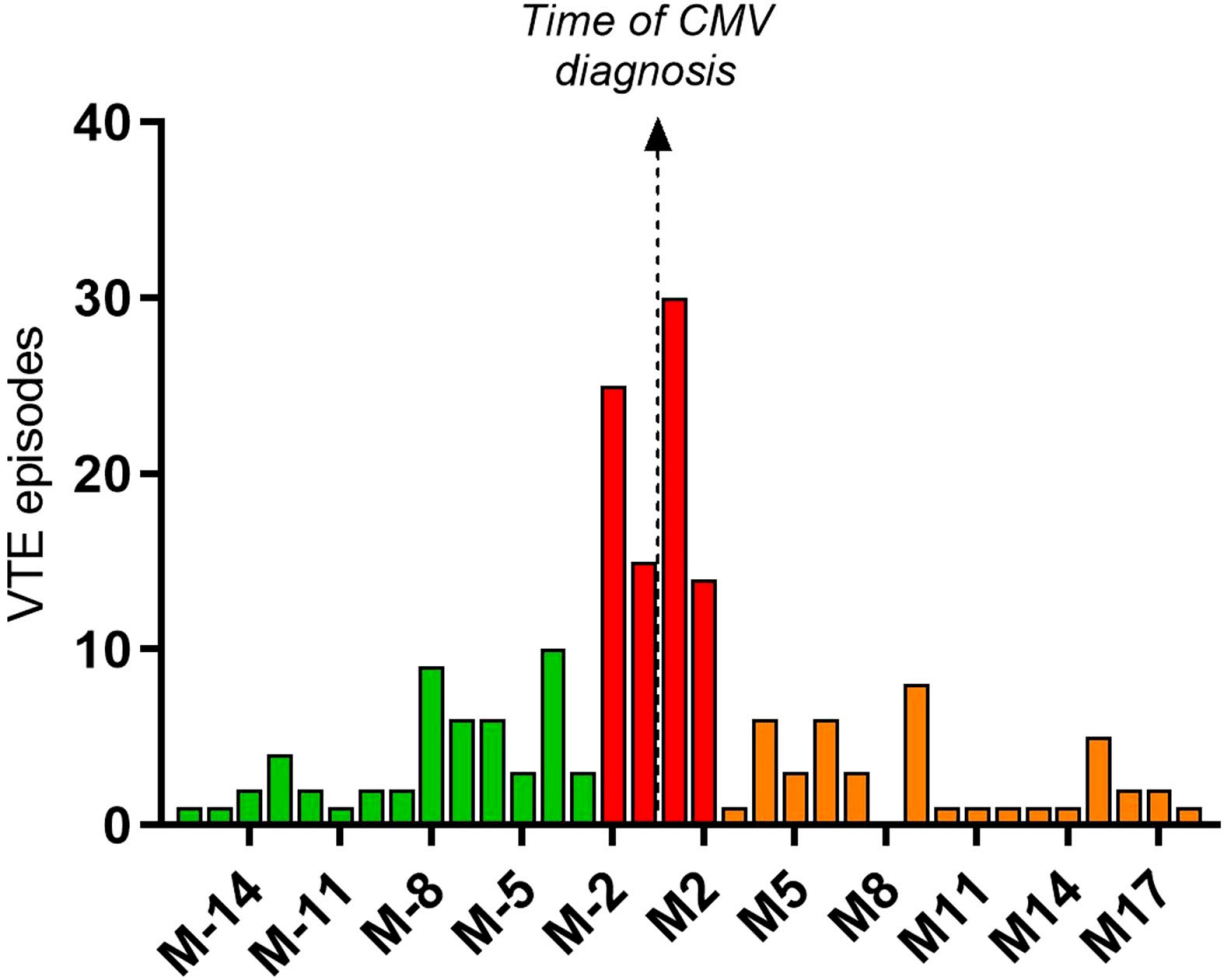
Distribution of the delay between the first CMV infection and the first VTE among the 179 patients with the two events.

For the 179 patients with both events, the median time of VTE diagnosis was 6 days before the CMV diagnosis. The complete representation of time to VTE diagnosis compared to time to CMV diagnosis is represented in **Figure 3**; **Table 3**. About half of the patients (n = 80) presented a diagnosis of VTE concurrently to CMV diagnosis (+/- 2 months, median time = 0 days after the diagnosis of CMV). In these patients, the diagnosis of VTE was deep vein thrombosis in 62.5% and pulmonary embolism in 23.9% (in 19.3%, the exact localization of VTE could not be retrieved). In patients who presented a VTE more than 2 months after CMV infection, 14 of them had available data regarding viremia follow-up: the mean duration was 105 days and 36% of them (5/14) presented a VTE while CMV viremia persisted.

**Table 3:** Description of the patients presenting with a VTE complication related to CMV infection. .

Parameter	All (n = 123)			VTE concurrent of CMV (n = 80)			VTE after CMV (n = 43)			
	NA	n	%	NA	n	%	NA	n	%	p-value
**Male recipient**	0	63	51.2	0	42	52.5	0	21	48.8	0.7099
**Retransplantation**	0	18	14.2	0	13	16.2	0	5	11.6	0.5982
**Kidney transplant alone**	0	120	94.5	0	78	97.5	0	42	97.7	>0.9999
**History of malignancy**	0	22	17.3	0	17	21.2	0	5	11.6	0.2236
**VTE before transplantation**	0	23	18.1	0	17	21.2	0	6	13.9	0.4667
**Positive recipient CMV serology**	0	88	69.3	0	57	71.2	0	31	72.1	>0.9999
**Positive donor CMV serology**	0	78	61.4	0	50	62.5	0	28	65.1	0.8457
**CMV Disease**	0	44	35.7	0	29	36.2	0	15	34.9	>0.9999
**Post-transplant CMV prophylaxis**	0	62	50.4	0	37	46.2	0	25	58.1	0.2575
**ECD**	9	64	54.2	8	42	58.3	1	22	52.4	>0.9999
**T-cell Depletant Induction**	0	93	73.2	0	59	73.7	0	34	79.1	>0.9999
**HLA incompatibilities > 4**	0	21	17.1	0	14	17.5	0	7	16.3	>0.9999
	NA	Mean	SD	NA	Mean	SD	NA	Mean	SD	**p-value**
**Recipient age (years)**	0	58.4	12.1	0	57.6	11.7	0	59.7	12.7	0.3374
**Recipient BMI (kg.m^2^)**	0	25.9	2.8	0	26.3	4.8	0	24.9	4.7	0.1127
**Time to the first CMV infection (days)**	0	161	146	0	121	152	0	114	124	0.9863
**Time to the first VTE (days)**	0	160	182	0	119	160	0	379	144	<0.0001

### Immunosuppressive drugs and occurrence of VTE

**Figure 4:**
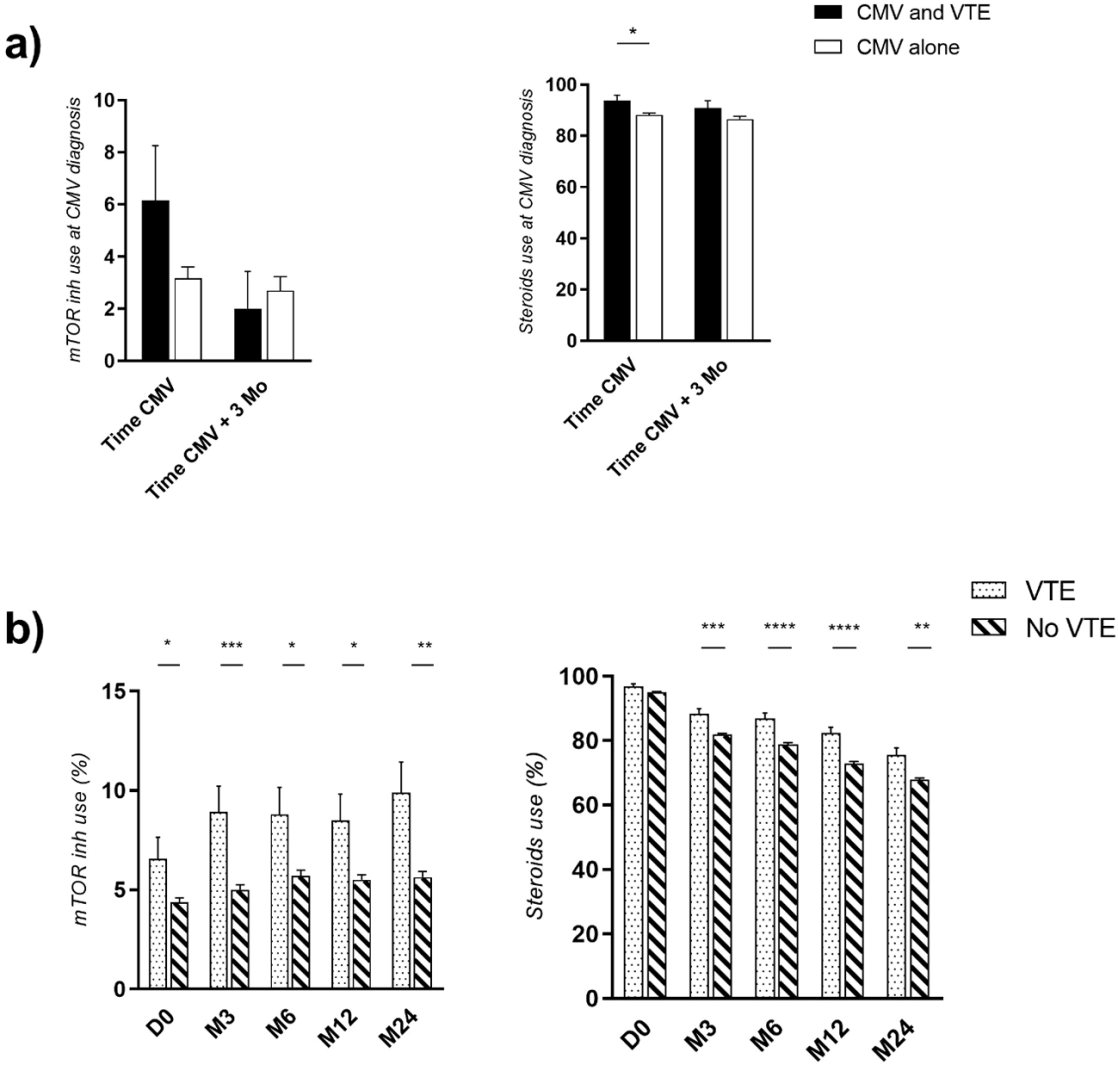
**(A)** Use of mTOR inhibitors and steroids among patients presenting a CMV infection without VTE compared to those with a VTE at the time of the CMV diagnosis and 3 months later **(B)**. Use of mTOR inhibitors and steroids depending on the occurrence or not of a VTE episode, in the subgroup of patients without CMV infection nor history/occurrence of neoplasia. * refers to p-value < 0.05, ** to p-value < 0.01, *** to p-value < 0.001 and **** to p-value < 0.0001.

Although complete follow-up data for maintenance therapy was missing in approximately 40% of our cohort—precluding time-varying analysis—we observed that among patients with complete follow-up, mTOR inhibitor use remained stable during the first 2 years post-transplantation in >90% of cases. In patients presenting a CMV infection, those who were complicated with a VTE episode received more mTOR inhibitors and steroids at the time of the CMV diagnosis compared to those without a VTE (6.15% vs 3.17%, p = 0.07 for mTOR inhibitors and 93.8 vs 88.1%, p = 0.04 for steroids), **Figure 4a**. However, in patients presenting neither CMV infection nor history/occurrence of neoplastic complication, the proportion of mTOR inhibitor and steroid use was also higher in patients who were complicated with a VTE than those who were not, (8.9 vs 5.0% at 3 months for the use of mTOR inhibitors, p = 0.0006 and 88.4 vs 81.9% for the use of steroids, p = 0.0003) **Figure 4b**. In summary, patients receiving mTOR inhibitors or steroids presented more VTE occurrence with or without CMV or neoplastic events.

## Discussion

Whilst an association between CMV infection and venous thrombosis has been the subject of multiple studies, particularly in immunocompetent patients, these almost always carry a bias linked to the serological diagnosis of CMV infection. We observed across a large multicenter cohort of kidney transplant recipients that CMV DNAemia is independently associated with a higher risk of a subsequent VTE event, using CMV viremia for the diagnosis of CMV infection rather than serological assessment. This risk was even higher in case of symptomatic CMV, but comparable for both primary infection and viral reactivation. To the best of our knowledge, an association between CMV and VTE in the field of transplantation has only been reported in a limited number of case reports (21–23, 31).

The specific role of immunosuppressive drugs in VTE occurrence warrants discussion, particularly since certain regimens may be prescribed in response to conditions that themselves increase VTE risk (e.g., mTOR inhibitors for CMV viremia or malignancy). Ideally, immunosuppressive regimens should have been modeled as time-varying covariates; however, this approach was not feasible due to substantial missing data for maintenance immunosuppression during follow-up. We conducted additional assessments showing that mTOR inhibitors and corticosteroid therapy appear associated with increased VTE risk. However, this probably does not confound the observed CMV-VTE association because only 5% of patients received mTOR inhibitors *de novo*; and among patients with complete follow-up data, immunosuppressive regimens remained stable during the first 2 years post-transplantation in the large majority of cases. The most likely reason is that mTOR inhibitors and steroid therapy adds an independent and cumulative risk for venous thrombotic complications, as shown several years ago by a large Canadian cohort (32).

An important question concerns the biological plausibility underlying the observed statistical association between CMV viremia and thrombosis. The heterogeneous temporal intervals in our cohort raise questions about causality: VTE events occurring months after documented CMV viremia are unlikely to be directly caused by the initial infection, even if some cases might be related to CMV recurrence and/or persistence. Conversely, the substantial proportion of VTE events occurring within close temporal proximity to CMV diagnosis likely reflects biological associations. Importantly, VTE events diagnosed shortly before documented CMV viremia may still be biologically linked, as subclinical viral replication and endothelial dysfunction (8, 33) likely precede detectable viremia by several days, while CMV detection depends on monitoring frequency. Recently, a large multicenter European cohort highlighted the association between CMV and microvascular inflammation in kidney transplant recipients, thus supports the involvement of CMV infection in endothelial dysfunction (13).

Our study has several limitations. Regarding our methods, we cannot exclude possible unobserved confounders and residual bias, as for a slight modification of our results since we excluded a small number of patients in multivariable analysis due to missing data. The lack of adjustment for potential time-varying confounders, such as immunosuppressive drug regimens, hospitalization duration, exact dosage and type of antithrombotic/anticoagulation drugs, or immunological factors such as hypogammaglobulinemia and lymphocyte count may have impacted our results. It is noteworthy that, despite the risk of VTE being associated stronger with CMV disease – often directly linked to the viremia load (10, 24) - compared to asymptomatic CMV infection, modelling CMV infection as a continuous parameter would have strengthen our results, While our analysis was stratified by transplant center — which accounts for systematic differences in center-specific practices and protocols — this approach does not fully address within-center variations in individual patient management over time. We acknowledge this as an inherent limitation of observational cohort studies with incomplete capture of time-dependent covariates. Additionally, variations in the timing and standardization of CMV monitoring across centers may have introduced detection bias and could represent a source of reverse causation, although CMV testing are less likely triggered by VTE occurrence.

In conclusion, we observed that CMV infection (both asymptomatic viremia and CMV disease) is associated with occurrence of venous thrombosis after kidney transplantation. Our findings may lead transplant physicians to adapt their anticoagulation prophylaxis after transplantation and suggest screening for CMV infection in cases of unexplained post-transplantation venous thrombosis.

## Data Availability

The data analyzed in this study is subject to the following licenses/restrictions: Data are available upon reasonable request to the corresponding author. Requests to access these datasets should be directed to christophe.masset@univ-nantes.fr.

## Supplementary material

Supplementary Table 1 Use of CMV prophylaxis in R-/D- after the surgery of transplantation depending on centers.

Supplementary Table 2 Use of CMV prophylaxis in R-/D+ after the surgery of transplantation depending on centers.

Supplementary Table 3 Use of CMV prophylaxis in R+/D- after the surgery of transplantation depending on centers.

Supplementary Table 4 Use of CMV prophylaxis in R+/D+ after the surgery of transplantation depending on centers.

Supplementary Table 5 Systematic use of antithrombotic prophylaxis after the surgery of transplantation depending on centers.

Supplementary Table 6 Results of the unadjusted cause-specific time-dependent Cox models stratified on centers studying the risk of venous thromboembolism in the first two years post-transplantation (n=15, 433, with 956 events observed during the follow-up).

Supplementary Table 7 Description of studied patients versus those excluded because of missing data (p-values are obtained using Chi-square test for categorical variables and Student t-test for continuous variables).

Supplementary Table 8 Sensitivity analysis in patients with positive recipient CMV serology. Results of the multivariable cause-specific time-dependent Cox model stratified on centers studying the risk of venous thromboembolism in the first two years post-transplantation (n=10, 028).

Supplementary Table 9 Sensitivity analysis in patients with positive donor CMV serology and negative recipient CMV serology. Results of the multivariable cause-specific time-dependent Cox model stratified on centers studying the risk of venous thromboembolism in the first two years post-transplantation (n=2, 576).

Supplementary Table 10 Sensitivity analysis in patients with kidney transplant. Results of the multivariable cause-specific time-dependent Cox model stratified on centers studying the risk of venous thromboembolism in the first two years post-transplantation (n=14, 510).

Supplementary Table 11 Results of the multivariable cause-specific time-dependent Cox model stratified on centers studying the risk of venous thromboembolism in the first two years post-transplantation considering surgical complications that may have occurred after the CMV infection (n=13, 943, 1, 490 recipients were removed because of missing data).
