# Supplementary material for: The risk of venous thromboembolism in kidney transplant recipients is enhanced following a cytomegalovirus infection
Source: Front Immunol. 2026 May 8;17:1816608. doi: 10.3389/fimmu.2026.1816608 (PMC13194019; doi:10.3389/fimmu.2026.1816608)
Supplement: Supplementary Table 1 — Use of CMV prophylaxis in R-/D- after the surgery of transplantation depending on centers. [file DataSheet1.docx]

**Supplementary Material**

**Table S1.** Use of CMV prophylaxis in R-/D- after the surgery of transplantation depending on centers.

| centre | CMV prophylaxis for R-D- | | | | | |
| --- | --- | --- | --- | --- | --- | --- |
|  | No | | Yes | | NA | |
|  | n | % | n | % | n | % |
| Center A | 204 | 63,4 | 112 | 34,8 | 6 | 1,9 |
| Center B | 336 | 82,4 | 40 | 9,8 | 32 | 7,8 |
| Center C | 361 | 97,3 | 8 | 2,2 | 2 | 0,5 |
| Center D | 948 | 87,0 | 137 | 12,6 | 5 | 0,5 |
| Center E | 24 | 5,9 | 371 | 91,8 | 9 | 2,2 |
| Center F | 25 | 39,1 | 33 | 51,6 | 6 | 9,4 |
| Center G | 44 | 97,8 | 1 | 2,2 | 0 | 0,0 |
| Center H | 77 | 60,6 | 25 | 19,7 | 25 | 19,7 |

**Table S2.** Use of CMV prophylaxis in R-/D+ after the surgery of transplantation depending on centers.

| Center | CMV prophylaxis for R-D+ | | | | | |
| --- | --- | --- | --- | --- | --- | --- |
|  | No | | Yes | | NA | |
|  | n | % | n | % | n | % |
| Center A | 4 | 1,3 | 309 | 98,7 | 0 | 0,0 |
| Center B | 54 | 11,1 | 423 | 86,9 | 10 | 2,1 |
| Center C | 21 | 5,6 | 351 | 93,9 | 2 | 0,5 |
| Center D | 42 | 6,1 | 645 | 93,6 | 2 | 0,3 |
| Center E | 8 | 1,8 | 422 | 96,6 | 7 | 1,6 |
| Center F | 1 | 1,0 | 101 | 96,2 | 3 | 2,9 |
| Center G | 5 | 15,6 | 27 | 84,4 | 0 | 0,0 |
| Center H | 18 | 12,9 | 82 | 58,6 | 40 | 28,6 |

**Table S3.** Use of CMV prophylaxis in R+/D- after the surgery of transplantation depending on centers.

| Center | CMV prophylaxis for R+D- | | | | | |
| --- | --- | --- | --- | --- | --- | --- |
|  | No | | Yes | | NA | |
|  | n | % | n | % | n | % |
| Center A | 12 | 1,8 | 637 | 97,4 | 5 | 0,8 |
| Center B | 495 | 73,8 | 153 | 22,8 | 23 | 3,4 |
| Center C | 450 | 93,8 | 24 | 5,0 | 6 | 1,3 |
| Center D | 141 | 15,5 | 764 | 84,0 | 5 | 0,6 |
| Center E | 12 | 1,5 | 796 | 96,8 | 14 | 1,7 |
| Center F | 4 | 2,8 | 138 | 95,8 | 2 | 1,4 |
| Center G | 53 | 85,5 | 8 | 12,9 | 1 | 1,6 |
| Center H | 191 | 30,9 | 295 | 47,7 | 133 | 21,5 |

**Table S4.** Use of CMV prophylaxis in R+/D+ after the surgery of transplantation depending on centers.

| Center | CMV prophylaxis for R+D+ | | | | | |
| --- | --- | --- | --- | --- | --- | --- |
|  | No | | Yes | | NA | |
|  | n | % | n | % | n | % |
| Center A | 12 | 1,5 | 763 | 97,5 | 8 | 1,0 |
| Center B | 748 | 74,4 | 210 | 20,9 | 47 | 4,7 |
| Center C | 533 | 91,4 | 44 | 7,6 | 6 | 1,0 |
| Center D | 81 | 10,8 | 664 | 88,7 | 4 | 0,5 |
| Center E | 32 | 2,4 | 1297 | 95,1 | 35 | 2,6 |
| Center F | 2 | 0,9 | 229 | 97,9 | 3 | 1,3 |
| Center G | 70 | 86,4 | 9 | 11,1 | 2 | 2,5 |
| Center H | 280 | 32,4 | 429 | 49,7 | 155 | 17,9 |

**Table S5.** Systematic use of antithrombotic prophylaxis after the surgery of transplantation depending on centers.

| Center | Antithrombotic prophylaxis post-transplantation |
| --- | --- |
| Center A | No |
| Center B | Yes |
| Center C | Yes |
| Center D | Yes |
| Center E | Yes |
| Center F | Yes |
| Center G | No |
| Center H | Yes |

**Table S6.** Results of the unadjusted cause-specific time-dependent Cox models stratified on centers studying the risk of venous thromboembolism in the first two years post-transplantation (n=15,433, with 956 events observed during the follow-up).

|  | **HR** | **95% CI** | **p-value** |
| --- | --- | --- | --- |
| **CMV Infection** | 1.84 | [1.43 ; 2.36] | <0.0001 |
| **Recipient age (years)** | 1.02 | [1.01 ; 1.02] | <0.0001 |
| **Male sex** | 0.94 | [0.83 ; 1.07] | 0.3493 |
| **Recipient BMI (kg/m²)** | 1.03 | [1.01 ; 1.04] | 0.0001 |
| **Re-transplantation** | 1.20 | [1.02 ; 1.40] | 0.0279 |
| **Kidney transplantation** | 0.84 | [0.64 ; 1.10] | 0.2026 |
| **Recurrent initial nephropathy** | 1.02 | [0.89 ; 1.18] | 0.7358 |
| **History of cardiovascular disease** | 1.54 | [1.35 ; 1.75] | <0.0001 |
| **History of hypertension** | 1.22 | [1.02 ; 1.46] | 0.0267 |
| **History of venous thromboembolism** | 2.73 | [2.27 ; 3.28] | <0.0001 |
| **History of malignancy** | 1.48 | [1.23 ; 1.78] | <0.0001 |
| **Positive recipient CMV serology** | 1.00 | [0.88 ; 1.15] | 0.9577 |
| **Donor age (years)** | 1.01 | [1.01 ; 1.02] | <0.0001 |
| **Male donor** | 1.00 | [0.88 ; 1.13] | 0.9679 |
| **Donor type** |  |  | <0.0001 |
| **ECD (vs. Living)** | 1.37 | [1.13 ; 1.67] |  |
| **SCD (vs. Living)** | 0.96 | [0.80 ; 1.17] |  |
| **Positive donor CMV serology** | 1.04 | [0.92 ; 1.19] | 0.5066 |
| **HLA-A-B-DR mismatches > 4** | 1.12 | [0.98 ; 1.27] | 0.0947 |
| **Depleting induction** | 1.06 | [0.93 ; 1.21] | 0.4027 |
| **CMV prophylaxis at transplantation** | 1.14 | [0.97 ; 1.35] | 0.1021 |
| **Positive anti class I** | 1.05 | [0.90 ; 1.21] | 0.5426 |
| **Positive anti class II** | 1.08 | [0.92 ; 1.26] | 0.3533 |
| **Delayed graft function** | 1.27 | [1.10 ; 1.46] | 0.0012 |
| **Surgical complication** | 1.69 | [1.46 ; 1.96] | <0.0001 |
| BMI, body mass index; CI, confidence interval; CMV, cytomegalovirus; ECD, expanded criteria donors; HLA, human leucocyte antigens; HR, hazard ratio; SCD, standard criteria donors; SD, standard deviation. The models were stratified on the center. | | | |

**Table S7.** Description of studied patients versus those excluded because of missing data (p-values are obtained using Chi-square test for categorical variables and Student t-test for continuous variables)

|  | **Whole sample (n=15,433)** | | | **Studied patients (n=13,943)** | | | **Excluded patients for missing data (n=1,490)** | | | **p-value** |
| --- | --- | --- | --- | --- | --- | --- | --- | --- | --- | --- |
|  | **NA** | **n** | **%** | **NA** | **n** | **%** | **NA** | **n** | **%** |  |
| **Male sex** | 0 | 9610 | 62.3 | 0 | 8661 | 62.1 | 0 | 949 | 63.7 | 0.2335 |
| **Re-transplantation** | 0 | 2596 | 16.8 | 0 | 2370 | 17.0 | 0 | 226 | 15.2 | 0.0727 |
| **Kidney transplantation** | 0 | 14510 | 94.0 | 0 | 13101 | 94.0 | 0 | 1409 | 94.6 | 0.3511 |
| **Recurrent initial nephropathy** | 0 | 4144 | 26.9 | 0 | 3749 | 26.9 | 0 | 395 | 26.5 | 0.7543 |
| **History of cardiovascular disease** | 0 | 5935 | 38.5 | 0 | 5420 | 38.9 | 0 | 515 | 34.6 | 0.0012 |
| **History of hypertension** | 0 | 12583 | 81.5 | 0 | 11507 | 82.5 | 0 | 1076 | 72.2 | <0.0001 |
| **History of venous thromboembolism** | 0 | 967 | 6.3 | 0 | 883 | 6.3 | 0 | 84 | 5.6 | 0.2925 |
| **History of malignancy** | 0 | 1636 | 10.6 | 0 | 1493 | 10.7 | 0 | 143 | 9.6 | 0.1856 |
| **Positive recipient CMV serology** | 0 | 10028 | 65.0 | 0 | 8964 | 64.3 | 0 | 1064 | 71.4 | <0.0001 |
| **Male donor** | 16 | 8790 | 57.0 | 11 | 7912 | 56.8 | 5 | 878 | 59.1 | 0.0841 |
| **Donor type** | 160 |  |  | 0 |  |  | 160 |  |  | <0.0001 |
| **ECD** |  | 4956 | 32.4 |  | 4474 | 32.1 |  | 482 | 36.2 |  |
| **Living** |  | 2367 | 15.5 |  | 2247 | 16.1 |  | 120 | 9.0 |  |
| **SCD** |  | 7950 | 52.1 |  | 7222 | 51.8 |  | 728 | 54.7 |  |
| **Positive donor CMV serology** | 0 | 8239 | 53.4 | 0 | 7443 | 53.4 | 0 | 796 | 53.4 | 0.9758 |
| **HLA-A-B-DR mismatches > 4** | 242 | 6897 | 45.4 | 0 | 6305 | 45.2 | 242 | 592 | 47.4 | 0.1320 |
| **Depleting induction** | 97 | 9074 | 59.2 | 27 | 8061 | 57.9 | 70 | 1013 | 71.3 | <0.0001 |
| **CMV prophylaxis at transplantation** | 607 | 9535 | 64.3 | 0 | 9149 | 65.6 | 607 | 386 | 43.7 | <0.0001 |
| **Positive anti class I** | 2190 | 5179 | 39.1 | 1743 | 4733 | 38.8 | 447 | 446 | 42.8 | 0.0118 |
| **Positive anti class II** | 2902 | 4930 | 39.3 | 2356 | 4564 | 39.4 | 546 | 366 | 38.8 | 0.7087 |
| **Delayed graft function** | 846 | 3567 | 24.5 | 0 | 3398 | 24.4 | 846 | 169 | 26.2 | 0.2800 |
| **At least one CMV infection** | 0 | 1756 | 11.4 | 0 | 1640 | 11.8 | 0 | 116 | 7.8 | <0.0001 |
|  | **NA** | **m** | **SD** | **NA** | **m** | **SD** | **NA** | **m** | **SD** | **p-value** |
| **Recipient age (years)** | 0 | 50.1 | 14.2 | 0 | 50.0 | 14.3 | 0 | 51.2 | 13.6 | 0.0010 |
| **Recipient BMI (kg/m²)** | 136 | 24.5 | 4.4 | 0 | 24.5 | 4.4 | 136 | 24.9 | 4.6 | 0.0003 |
| **Donor age (years)** | 49 | 50.9 | 16.4 | 0 | 50.8 | 16.5 | 49 | 51.5 | 16.2 | 0.1195 |
| BMI, body mass index; CMV, cytomegalovirus; ECD, expanded criteria donors; HLA, human leucocyte antigens; NA: not available (missing); SCD, standard criteria donors; SD, standard deviation. | | | | | | | | | | |

**Table S8.** Sensitivity analysis in patients with positive recipient CMV serology. Results of the multivariable cause-specific time-dependent Cox model stratified on centers studying the risk of venous thromboembolism in the first two years post-transplantation (n=10,028).

|  | **HR** | **95% CI** | **p-value** |
| --- | --- | --- | --- |
| **CMV infection** |  |  | 0.0035 |
| *CMV DNAemia* | 1.66 | [1.16 ; 2.39] |  |
| *Symptomatic CMV* | 1.94 | [1.12 ; 3.36] |  |
| **Year of transplantation > 2010** | 0.80 | [0.67 ; 0.96] | 0.0133 |
| **Recipient age (years)** | 1.01 | [1.00 ; 1.02] | 0.0111 |
| **Recipient BMI (kg/m²)** | 1.02 | [1.00 ; 1.04] | 0.0890 |
| **Retransplantation** | 1.05 | [0.84 ; 1.30] | 0.6870 |
| **History of cardiovascular disease** | 0.90 | [0.74 ; 1.09] | 0.2765 |
| **History of hypertension** | 1.01 | [0.81 ; 1.27] | 0.9224 |
| **History of venous thromboembolism** | 2.86 | [2.21 ; 3.70] | <0.0001 |
| **History of malignancy** | 1.17 | [0.91 ; 1.51] | 0.2303 |
| **Donor age (years)** | 1.00 | [0.99 ; 1.01] | 0.4398 |
| **Donor type** |  |  | 0.1729 |
| **ECD (vs. Living)** | 0.83 | [0.62 ; 1.12] |  |
| **SCD (vs. Living)** | 0.82 | [0.64 ; 1.05] |  |
| **HLA-A-B-DR mismatches > 4** | 1.07 | [0.90 ; 1.26] | 0.4609 |
| **CMV prophylaxis treatment at transplantation** | 1.23 | [0.91 ; 1.66] | 0.1835 |
| **Delayed graft function** | 1.16 | [0.96 ; 1.40] | 0.1328 |
| **Surgical complication** | 1.74 | [1.44 ; 2.10] | <0.0001 |
| BMI, body mass index; CI, confidence interval; CMV, cytomegalovirus; ECD, expanded criteria donors; HLA, human leucocyte antigens; HR, hazard ratio; SCD, standard criteria donors; SD, standard deviation. | | | |

**Table S9.** Sensitivity analysis in patients with positive donor CMV serology and negative recipient CMV serology. Results of the multivariable cause-specific time-dependent Cox model stratified on centers studying the risk of venous thromboembolism in the first two years post-transplantation (n=2,576).

|  | **HR** | **95% CI** | **p-value** |
| --- | --- | --- | --- |
| **CMV infection** |  |  | 0.0046 |
| *CMV DNAemia* | 2.37 | [1.23 ; 4.54] |  |
| *Symptomatic CMV* | 2.54 | [1.26 ; 5.13] |  |
| **Year of transplantation > 2010** | 0.70 | [0.50 ; 0.98] | 0.0380 |
| **Recipient age (years)** | 1.01 | [0.99 ; 1.03] | 0.1910 |
| **Recipient BMI (kg/m²)** | 0.99 | [0.94 ; 1.03] | 0.5060 |
| **Retransplantation** | 1.55 | [1.01 ; 2.37] | 0.0436 |
| **History of cardiovascular disease** | 1.27 | [0.90 ; 1.80] | 0.1659 |
| **History of hypertension** | 1.55 | [0.95 ; 2.55] | 0.0810 |
| **History of venous thromboembolism** | 1.82 | [1.05 ; 3.13] | 0.0323 |
| **History of malignancy** | 1.04 | [0.67 ; 1.63] | 0.8549 |
| **Donor age (years)** | 1.01 | [0.99 ; 1.03] | 0.2974 |
| **Donor type** |  |  | 0.6466 |
| **ECD (vs. Living)** | 0.94 | [0.55 ; 1.61] |  |
| **SCD (vs. Living)** | 1.22 | [0.71 ; 2.11] |  |
| **HLA-A-B-DR mismatches > 4** | 1.28 | [0.92 ; 1.79] | 0.1367 |
| **CMV prophylaxis treatment at transplantation** | 1.36 | [0.65 ; 2.85] | 0.4204 |
| **Delayed graft function** | 1.15 | [0.80 ; 1.66] | 0.4564 |
| **Surgical complication** | 1.28 | [0.86 ; 1.89] | 0.2221 |
| BMI, body mass index; CI, confidence interval; CMV, cytomegalovirus; ECD, expanded criteria donors; HLA, human leucocyte antigens; HR, hazard ratio; SCD, standard criteria donors; SD, standard deviation. | | | |

**Table S10.** Sensitivity analysis in patients with kidney transplant. Results of the multivariable cause-specific time-dependent Cox model stratified on centers studying the risk of venous thromboembolism in the first two years post-transplantation (n=14,510).

|  | **HR** | **95% CI** | **p-value** |
| --- | --- | --- | --- |
| **CMV infection** |  |  | 0.0001 |
| *CMV DNAemia* | 1.66 | [1.22 ; 2.26] |  |
| *Symptomatic CMV* | 2.07 | [1.37 ; 3.12] |  |
| **Year of transplantation > 2010** | 0.82 | [0.70 ; 0.94] | 0.0065 |
| **Recipient age (years)** | 1.01 | [1.01 ; 1.02] | 0.0001 |
| **Recipient BMI (kg/m²)** | 1.01 | [1.00 ; 1.03] | 0.0809 |
| **Retransplantation** | 1.22 | [1.02 ; 1.46] | 0.0280 |
| **History of cardiovascular disease** | 0.99 | [0.84 ; 1.15] | 0.8575 |
| **History of hypertension** | 1.06 | [0.87 ; 1.28] | 0.5730 |
| **History of venous thromboembolism** | 2.56 | [2.07 ; 3.17] | <0.0001 |
| **History of malignancy** | 1.23 | [1.01 ; 1.50] | 0.0397 |
| **Donor age (years)** | 1.00 | [0.99 ; 1.01] | 0.7354 |
| **Donor type** |  |  | 0.2965 |
| **ECD (vs. Living)** | 0.91 | [0.72 ; 1.15] |  |
| **SCD (vs. Living)** | 0.86 | [0.70 ; 1.06] |  |
| **HLA-A-B-DR mismatches > 4** | 1.10 | [0.95 ; 1.27] | 0.1902 |
| **CMV prophylaxis treatment at transplantation** | 1.15 | [0.96 ; 1.38] | 0.1174 |
| **Delayed graft function** | 1.10 | [0.94 ; 1.29] | 0.2351 |
| **Surgical complication** | 1.57 | [1.34 ; 1.84] | <0.0001 |
| BMI, body mass index; CI, confidence interval; CMV, cytomegalovirus; ECD, expanded criteria donors; HLA, human leucocyte antigens; HR, hazard ratio; SCD, standard criteria donors; SD, standard deviation. | | | |

**Table S11.** Results of the multivariable cause-specific time-dependent Cox model stratified on centers studying the risk of venous thromboembolism in the first two years post-transplantation considering surgical complications that may have occurred after the CMV infection (n=13,943, 1,490 recipients were removed because of missing data).

|  | **HR** | **95% CI** | **p-value** |
| --- | --- | --- | --- |
| **CMV infection** |  |  | 0.0011 |
| *CMV DNAemia* | 1.58 | [1.17 ; 2.13] |  |
| *Symptomatic CMV* | 1.93 | [1.29 ; 2.92] |  |
| **Transplantation year after 2010** | 0.84 | [0.73 ; 0.97] | 0.0154 |
| **Recipient age (years)** | 1.01 | [1.00 ; 1.02] | 0.0009 |
| **Recipient BMI (kg/m²)** | 1.01 | [1.00 ; 1.03] | 0.1041 |
| **Retransplantation** | 1.18 | [0.99 ; 1.41] | 0.0613 |
| **History of cardiovascular disease** | 1.03 | [0.88 ; 1.20] | 0.7322 |
| **History of hypertension** | 1.10 | [0.91 ; 1.33] | 0.3227 |
| **History of venous thromboembolism** | 2.50 | [2.03 ; 3.08] | <0.0001 |
| **History of malignancy** | 1.21 | [0.99 ; 1.47] | 0.0579 |
| **Donor age (years)** | 1.00 | [0.99 ; 1.01] | 0.7031 |
| **Donor type** |  |  | 0.4264 |
| *ECD (vs. Living)* | 0.93 | [0.73 ; 1.17] |  |
| *SCD (vs. Living)* | 0.90 | [0.73 ; 1.11] |  |
| **HLA-A-B-DR mismatches > 4** | 1.12 | [0.98 ; 1.28] | 0.1095 |
| **CMV prophylaxis at transplantation** | 1.15 | [0.96 ; 1.37] | 0.1207 |
| **Delayed graft function** | 1.09 | [0.93 ; 1.27] | 0.2853 |
| **Surgical complication** | 1.58 | [1.36 ; 1.85] | <0.0001 |
| BMI, body mass index; CI, confidence interval; CMV, cytomegalovirus; ECD, expanded criteria donors; HLA, human leucocyte antigens; HR, hazard ratio; SCD, standard criteria donors; SD, standard deviation. | | | |
